# M. Galippe

**Published:** 1887-10

**Authors:** 


**M. Galippe** has related to the Paris Academy of Medicine the discovery of a new fungus composed of tubes and spores of mycelium developed in the human saliva. It belongs to the Moniliæ family, and M. Galippe has given it the name of *Monilia sputicola.*
—*The Microscope.*

